# Clinical analysis of the effect of helicobacter pylori infection on immune function in children with peptic ulcer

**DOI:** 10.12669/pjms.40.6.7820

**Published:** 2024-07

**Authors:** Yongnan Teng, Qingwei Dong, Sisi Zhang, Songsong Chen, Chen Li

**Affiliations:** 1 Yongnan Teng, Department of Gastroenterology, Baoding Hospital, Beijing Children’s Hospital Affiliated to Capital Medical University, Baoding, Hebei, 071000, P.R. China; Key Laborary of Clinical, Research on Respiratory Digestive Disease, Hebei Baoding, 071000, China; 2 Qingwei Dong, Department of Gastroenterology, Baoding Hospital, Beijing Children’s Hospital Affiliated to Capital Medical University, Baoding, Hebei, 071000, P.R. China; Key Laborary of Clinical, Research on Respiratory Digestive Disease, Hebei Baoding, 071000, China; 3 Sisi Zhang, Department of Gastroenterology, Baoding Hospital, Beijing Children’s Hospital Affiliated to Capital Medical University, Baoding, Hebei, 071000, P.R. China; Key Laborary of Clinical, Research on Respiratory Digestive Disease, Hebei Baoding, 071000, China; 4 Songsong Chen, Department of Gastroenterology, Baoding Hospital, Beijing Children’s Hospital Affiliated to Capital Medical University, Baoding, Hebei, 071000, P.R. China; Key Laborary of Clinical, Research on Respiratory Digestive Disease, Hebei Baoding, 071000, China; 5 Chen Li, Department of Gastroenterology, Baoding Hospital, Beijing Children’s Hospital Affiliated to Capital Medical University, Baoding, Hebei, 071000, P.R. China; Key Laborary of Clinical, Research on Respiratory Digestive Disease, Hebei Baoding, 071000, China

**Keywords:** Helicobacter pylori, Peptic ulcer, T Lymphocyte subgroup, Immunoglobulin

## Abstract

**Objective:**

To study whether children with peptic ulcer would have abnormalities in cellular and humoral immune functions, and whether Helicobacter pylori (Hp) infection would affect the immune function of children with peptic ulcer.

**Methods:**

This is a retrospective study. The subjects of study were 72 children with diagnosed and cured peptic ulcer (ulcer group), and 50 healthy children with physical examination (control group) at Baoding Hospital, Beijing Children’s Hospital Affiliated to Capital Medical University from June 2020 to December 2022. Further detection was conducted on T lymphocyte subsets (CD3+, CD4+, CD8+, and CD4+/CD8+ ratio) and immunoglobulin levels.

**Results:**

Of the 72 children with peptic ulcer, 53(73.6%) were positive for Hp (Hp-positive group) and 19 (26.4%) were negative (Hp-negative group). The levels of CD3^+^, CD4^+^, and CD4^+^/CD8^+^ ratio in the control group were significantly higher than those in the ulcer group, with statistically significant difference (P<0.05); while the level of IgG in the control group was lower than that in the ulcer group, with statistically significant difference (P<0.05). Meanwhile, there were statistically significant differences in that the levels of CD3^+^, CD4^+^ and CD8^+^ were increased in Hp-positive group than those in Hp-negative group before treatment (P<0.05); while CD4^+^/CD8^+^ ratio was lower in the former group than that in the latter group, with statistically significant difference (P<0.05).

**Conclusion:**

Hp infection can induce the elevation of T lymphocyte subsets. The development of peptic ulcer has an intimate association with the disorder of cellular and humoral immune functions.

## INTRODUCTION

Helicobacter pylori (Hp) infection affects nearly half of the world’s population,^1^ which has become one of the major public health challenges globally, especially in developing countries.^2^ It is a chronic disease that occurs commonly in children^3^, which has been accepted to be an important cause of chronic gastritis and peptic ulcer in this group of population.^4^ It is now recognized that Hp infection can affect the immune response of human body, and immune factors function significantly in the occurrence and development of peptic ulcer.^5^-^7^ However, it remains unclear with respect to the immunological mechanism of peptic ulcer, especially in childhood.^8^ In order to identify whether children with peptic ulcer would have abnormalities in cellular and humoral immune functions, and whether Hp infection would affect the immune function of children with peptic ulcer, these pro-inflammatory cytokines are necessary and sufficient to induce villus damage and disrupt intestinal barrier function, this study detected the levels of T lymphocyte subsets and immunoglobulins in peripheral blood of children with peptic ulcer in our hospital. The report is as follows.

## METHODS

This is a retrospective study. A total of 72 children with peptic ulcer who were diagnosed by electronic gastroscopy and cured in Baoding Hospital, Beijing Children’s Hospital Affiliated to Capital Medical University from June 2020 to December 2022. These children were classified into the ulcer group, including 43 boys and 29 girls, with an average age of (10.5±2.5) years. Simultaneously, 50 healthy children who underwent physical examination in our hospital were enrolled in the control group, including 29 boys and 21 girls, with an average age of (10.0±2.4) years. There was no statistical significance between the two groups in the comparison of gender and age (P> 0.05).

### Ethical Approval

The study was approved by the Institutional Ethics Committee of Baoding Hospital, Beijing Children’s Hospital Affiliated to Capital Medical University (No.:2020035; date: June 16,2020), and written informed consent was provided by family members of the enrolled children.

### Diagnostic criteria of Hp infection

The diagnosis of Hp infection was determined based on endoscopic pathological staining, rapid urease test and/or 13C-urea breath test.^9^

### Inclusion Criteria:

- Children aged 6-16 years old;
- Patients with abdominal pain, nausea and other digestive system symptoms;
- Hp-positive patients diagnosed by endoscopic pathological staining and rapid urease test;
- Patients successfully followed up for one year.

### Exclusion criteria:

- Children with endocrine, genetic and metabolic diseases, tumors, allergic diseases, immune diseases or mental diseases.
- Children who had been diagnosed with chronic diseases or acute diseases in the past one month.
- Children whose ulcer was not cured or whose Hp was reexamined to be positive after treatment.
- Children under six years old.
- Children with false positive HP infection.

### Gastroscopy

All children enrolled in this study were subjected to gastroscopy by trained and qualified staff using Olympus Q290 electronic gastroscope. The enrolled children were examined with electronic gastroscope before treatment and after treatment with symptoms disappeared (at least one month after treatment).

### Therapeutic scheme

Children with Hp-positive peptic ulcer were given amoxicillin at the dose of 20 mg/kg.d (twice a day orally for two weeks), with the maximum dose of 0.5 g/time; clarithromycin at the dose of 20 mg/kg.d (twice a day orally for two weeks), with the maximum dose of 0.5 g/time; omeprazole at the dose of 0.6-1.0 mg/kg.d (twice a day orally for four weeks), with the maximum dose of 20 mg/time; and Colloidal Bismuth Subcitrate at the dose of 6-8 mg/kg.d (twice a day orally for four weeks). Children with Hp-negative peptic ulcer was treated with omeprazole and Colloidal Bismuth Subcitrate at the same dose as above.

### Sampling

An amount of two ml of venous blood were collected from each child on an empty stomach in the morning via the purple-cap tube and the yellow-cap tube, respectively. The blood samples were placed at 4°C-20°C for 30 minutes, and centrifuged at 3000 r/min for 10 min, after which the serum from blood in the yellow-cap tube was collected for determination.

Detection of T lymphocyte subsets (CD3^+^, CD4^+^, CD8^+^, and CD4^+^/CD8^+^ ratio) and immunoglobulin levels: The detection of T lymphocyte subsets and immunoglobulins was carried out by qualified personnel from Baoding Key Laboratory of Clinical Research on Children’s Respiratory and Digestive Diseases, Hebei Province. McAb-immunofluorescence was adopted for detecting T lymphocyte subsets using reagents from QuantoBio by the experimental instrument of MindyayBriCyte E6 flow cytometer. Immunoglobulins were detected by immunoturbidimetry using reagents and test instrument (IMMAGE800) purchased from Beckman Coulter.

### Statistical analysis

SPSS22.0 statistical software package was used for statistical analysis of this study; the confidence interval is 95%. The measurement data were expressed by (*χ̅*±*S*), and compared using analysis of variance and t-test between groups. There was Statistically significant difference at P<0.05.

## RESULTS

Of the 72 children with peptic ulcer, 65 children had duodenal ulcer (90.3%) and seven had gastric ulcer (9.7%). Of the 72 children with peptic ulcer, 53 (73.6%) were positive for Hp (Hp-positive group) and 19 (26.4%) were negative (Hp-negative group). In Hp-positive group, there were 31 boys and 22 girls; and in Hp-negative group, there were 12 boys and seven girls, with an average age of (10.4±2.5) years and (10.6±2.6) years, respectively. Besides, there were 50 children in the control group, including 29 boys and 21 girls, with an average age of (10.0±2.4) years. There was no statistical significance in gender and age among the three groups (P>0.05; Table-I).

**Table-I:** Data of Hp infection.

Groups	Cases (n)	Boys (n)	Girls (n)	Age (years)
Hp-positive group	53 (73.6%)	31	22	10.4±2.5
Hp-negative group	19 (26.4%)	12	7	10.6±2.6
Control group	50	29	21	10.0±2.4

Analysis of T lymphocyte subsets and immunoglobulin levels in control group and ulcer group. As shown in Table-II, the levels of CD3^+^, CD4^+^, and CD4^+^/CD8^+^ ratio in the control group were significantly higher than those in the ulcer group, with statistically significant difference (P<0.05); while the level of IgG in the control group was lower than that in the ulcer group, with statistically significant difference (P<0.05). In addition, there was no statistically significant difference in the levels of CD8^+^, IgA and IgM between the two groups (P>0.05).

In Table-III, there were statistically significant differences that the levels of CD3^+^, CD4^+^ and CD8^+^ were increased in Hp-positive group than those in Hp-negative group before treatment (P<0.05); while CD4^+^/CD8^+^ ratio was lower in the former group than that in the latter group, with statistically significant difference (P<0.05). However, there was no statistically significant difference in the comparison of IgA, IgG and IgM between Hp-positive group and Hp-negative group before treatment. There was no statistically significant difference in the comparison of CD3^+^, CD4^+^, CD8^+^, CD4^+^/CD8^+^ ratio, IgA, IgG and IgM between Hp-positive group and Hp-negative group after treatment (Table-IV).

**Table-II:** T lymphocyte subsets and immunoglobulin levels in control group and ulcer group.

Groups	Cases (n)	CD3^+^ (×10^6^/L)	CD4^+^ (×10^6^/L)	CD8^+^ (×10^6^/L)	CD4^+^/CD8^+^	IgA (g/L)	IgM (g/L)	IgG (g/L)
Control group	50	1277±369	945±198	385±153	2.99±1.77	2.28±0.55	1.01±0.23	9.57±1.04
Ulcer group	72	1110±454	691±235	389±153	2.06±1.12	2.34±0.68	1.10±0.35	11.65±1.46
t value		2.237	6.257	0.149	3.288	0.476	1.725	9.174
P value		0.027	0.000	>0.05	0.002	>0.05	>0.05	0.000

**Table-III:** T lymphocyte subsets and immunoglobulin levels in Hp-positive group and Hp-negative group before treatment.

Groups	Cases (n)	CD3^+^ (×10^6^/L)	CD4^+^ (×10^6^/L)	CD8^+^ (×10^6^/L)	CD4^+^/CD8^+^	IgA (g/L)	IgM (g/L)	IgG (g/L)
Hp-positive group	53	1193±467	725±239	435±141	1.84±0.81	2.28±0.58	1.13±0.26	11.50±1.28
Hp-negative group	19	877±322	594±200	262±108	2.66±1.59	2.51±0.88	1.03±0.51	12.07±1.85
t value		2.715	2.145	4.854	2.165	1.055	0.801	1.243
P value		0.008	0.035	0.000	0.042	>0.05	>0.05	>0.05

**Table-IV:** T-lymphocyte subsets and immunoglobulin levels in Hp-positive group and Hp-negative group after treatment.

Groups	Cases (n)	CD3^+^ (×10^6^/L)	CD4^+^ (×10^6^/L)	CD8^+^ (×10^6^/L)	CD4^+^/CD8^+^	IgA (g/L)	IgM (g/L)	IgG (g/L)
Hp-positive group	53	1138±412	896±178	389±138	2.71± 1.52	2.27±0.48	1.11±0.24	10.56±1.46
Hp-negative group	19	1193±467	725±239	435±141	1.84±0.81	2.28±0.58	1.13±0.26	11.50±1.28
t value		0.250	0.330	0.122	0.606	0.188	1.755	0.056
P value		>0.05	>0.05	>0.05	>0.05	>0.05	>0.05	>0.05

## DISCUSSION

T lymphocyte subsets and immunoglobulins are integral parts of the mechanism of immune defense *in vivo*. There is a mild-to-moderate positive and negative correlation between active innate and adaptive immune indexes secreted by gastric mucosa in patients with peptic ulcer.^10^ Immunosuppression was reported to be detected in the cellular immunity of patients with peptic ulcer.^11^ In our study, the levels of CD3^+^, CD4^+^, and CD4^+^/CD8^+^ ratio in children with peptic ulcer were lower than those in healthy children, which were consistent with previous reports. It is explained that considering the disorder of immune function in children with peptic ulcer, the changes of T lymphocyte subset and immunoglobulin levels may be associated strongly with children with peptic ulcer.

Hp infection can cause chronic inflammation of the digestive system, the host’s immune response, however, can rarely clear Hp.^12^ About 15% of the infected individuals may develop severe gastric and duodenal lesions.^13^ However, it is still not fully clarified and remains controversial with regard to the pathological mechanism of Hp on gastric mucosa and its role in the pathogenesis of peptic ulcer.

According to previous research^14^, T-lymphocytes play an important role in the immunopathogenesis of digestive system diseases associated with Hp infection. The increase of CD4^+^T lymphocyte level has a close relationship with the injury of gastric mucosa caused by Hp infection.^15^ Moreover, CD4+T cells may function significantly in regulating or inhibiting CD8^+^T cells, and CD8^+^T cells may mediate more serious diseases in the absence of CD4^+^T cells.^16^ There would be obvious increase in the levels of CD4^+^ and CD8^+^T lymphocytes in peripheral blood of children with Hp infection.^17^ Besides, CD4^+^T lymphocyte count in patients with Hp infection-associated peptic ulcer was higher than that in children without infection.^18^ Similar to previous reports, in this study, the levels of CD3^+^, CD4^+^ and CD8^+^ in children with Hp-positive peptic ulcer were higher than those in children with Hp-negative peptic ulcer. As for corresponding reason, children with Hp infection may have changes in levels of T lymphocytes and produce strong T lymphocyte response, thereby leading to the presence of pathological reactions, such as inflammatory damage and peptic ulcer.

Hp infection can cause strong humoral immune response.^19^ Children with peptic ulcer may develop humoral immune disorder characterized by increased lymphocyte levels.^13^ In our study, the level of IgG in children with peptic ulcer was higher than that in healthy children, yet without significant difference in IgA, IgG and IgM levels between children with Hp-positive ulcer and those with Hp-negative ulcer. It can be understood that children with peptic ulcer may have disorders of the humoral immune function, while Hp infection has none significant impact on humoral immunity of children with peptic ulcer.

Results of this study indicated that after treatment, there was no statistical difference in the levels of T lymphocyte subsets and immunoglobulins between children with Hp-positive peptic ulcer and those with Hp-negative peptic ulcer. It is suggested that the effect of Hp infection on human body may disappear soon after medication, and the autoimmune function *in vivo* can respond in a timely manner.

### Limitations of the study

It includes as small sample size, single-center design, and no further subgroup analysis on the severity of peptic ulcer. In view of this, further improvements will be made in future research to make more scientific research results.

## CONCLUSIONS

Hp infection can induce the elevation of T lymphocyte subsets. The development of peptic ulcer has an intimate association with the disorder of cellular and humoral immune functions.

### Authors’ Contributions:

**YT** and **QD:** Carried out the studies, participated in collecting data, and drafted the manuscript, and are responsible and accountable for the accuracy and integrity of the work.

**SZ** and **SC:** Performed the statistical analysis and participated in its design.

**CL:** Participated in acquisition, analysis, or interpretation of data and draft the manuscript. All authors read and approved the final manuscript.
